# Stability Risk Assessment of Underground Rock Pillars Using Logistic Model Trees

**DOI:** 10.3390/ijerph19042136

**Published:** 2022-02-14

**Authors:** Ning Li, Masoud Zare, Congke Yi, Rafael Jimenez

**Affiliations:** 1 School of Mining Engineering, Anhui University of Science and Technology, Huainan 232001, China; nli@aust.edu.cn; 2 United Consulting Group Ltd., Norcross, GA 30071, USA; 3 ETSI Caminos, Canales y Puertos, Technical University of Madrid, 28040 Madrid, Spain; congke.yi@alumnos.upm.es (C.Y.); rafael.jimenez@upm.es (R.J.)

**Keywords:** rock pillar, Logistic Model Trees (LMT), stability prediction, cross-validation

## Abstract

Pillars are important structural elements that provide temporary or permanent support in underground spaces. Unstable pillars can result in rock sloughing leading to roof collapse, and they can also cause rock burst. Hence, the prediction of underground pillar stability is important. This paper presents a novel application of Logistic Model Trees (LMT) to predict underground pillar stability. Seven parameters—pillar width, pillar height, ratio of pillar width to height, uniaxial compressive strength of rock, average pillar stress, underground depth, and Bord width—are employed to construct LMTs for rock and coal pillars. The LogitBoost algorithm is applied to train on two data sets of rock and coal pillar case histories. The two models are validated with (i) 10-fold cross-validation and with (ii) another set of new case histories. Results suggest that the accuracy of the proposed LMT is the highest among other common machine learning methods previously employed in the literature. Moreover, a sensitivity analysis indicates that the average stress, *p*, and the ratio of pillar width to height, *r*, are the most influential parameters for the proposed models.

## 1. Introduction

Pillars are important structural elements in rock and coal underground mining since they can provide temporary or permanent support for tunneling and mining work [1,2,3]. To maximize the extraction rate of mineral resources, dimensions (width and height) of underground pillars must be confined to a certain range, which should still assure stability and safe working conditions throughout the entire life of such projects [4]. Unstable pillars can result in rock cracking, causing roof collapse, and they could even trigger rock bursts when large amounts of accumulated elastic energy are released [3,5]. Increases of mining depth can lead to more frequent pillar instability and failure events; it is therefore expected that new design challenges must be addressed so that new methods to assess rock pillar stability be discussed and developed.

The most common parameter in pillar design and stability estimation is the factor of safety (*FoS*), which can be calculated as the ratio of pillar strength to applied pillar stress. Two main methods —the tributary area theory, and numerical simulations—are often employed for pillar stress calculation [3,6]; whereas several empirical methods have been developed for pillar strength estimation, as listed in Table 1. Three basic ‘effects’ related with the ratio of pillar height to width—the linear shape effect, size effect, and power shape effect—were summarized by Lunder [6]; similarly, Lunder [6] worked out an empirical equation to assess pillar strength, considering confined and unconfined strength and different ratios of pillar height to width. Similarly, Ahmad and Al-Shayea et al. [7] proposed new coal pillar strength formulae that consider the interface friction between the roof/floor and pillar, which also have a linear and power shape effect.

Theoretically, a rock or coal pillar is predicted as ‘stable’ when the value of *FoS* is greater than 1; otherwise, it is considered ‘unstable’. However, such crisp boundaries are often unreliable, as unstable pillars often occurs when the *FoS* value is above 1 (Lunder [6]), and uncertainties in the ‘capacity’ (strength or resisting force) and ‘demand’ (stress or disturbing force) of a pillar lead to the *FoS* being described as a statistical distribution.

Numerical simulation has also been employed to evaluate pillar stability. For instance, York [15] and Mortazavi and Hassani et al. [16] employed FLAC^2D^ to estimate the stability of deep-level pillars considering pillar deformations under natural loading conditions. Li and Li et al. [17] employed FLAC^3D^ to obtain the minimum required thickness of crown pillars under sea water pressure; and Martin and Maybee [18] conducted two-dimensional finite element analyses using Hoek-Brown parameters for pillar strength. Similarly, the finite element method and the discrete fracture network (DFN) approach were integrated by Deng and Yue et al. [19] and Elmo and Stead [20] to study the failure of jointed pillars. However, although such numerical methods are powerful tools to model pillar behavior, they have limitations due to the complexity of the models involved and the need for specific calibration.

Many machine learning algorithms have been applied to predict pillar stability. For instance, Tawadrous and Katsabanis [21] employed Artificial Neural Networks (ANN). Recio-Gordo and Jimenez [22] presented a probabilistic model based on the theory of linear classifiers that can be used to make probabilistic predictions of pillar behavior in longwall and retreat room and pillar mining. Zhou and Li et al. [2] employed Fisher Discriminant Analysis (FDA) and Support Vector Machines (SVM), whereas Wattimena and Kramadibrata et al. [23] and Wattimena [4] applied logistic regression algorithms. Ghasemi and Ataei et al. [24] and Ghasemi and Ataei et al. [25] developed logistic regression and fuzzy logic algorithms in room-and-pillar coal mines. Zhou and Li et al. [3] employed six machine learning algorithms to evaluate the stability of a rock pillar, and Ghasemi and Kalhori et al. [26] compared Decision Tree (J48) and SVM algorithms for hard rock pillar stability assessment. Mohanto and Deb [27] developed a plastic damage index for assessing rib pillar stability in underground metal mines using multi-variate regression and artificial neural network techniques. Ahmad and Al-Shayea et al. [7] employed random trees and C4.5 decision trees for underground pillar prediction, and Liang and Luo et al. [28] used new GBDT, XGBoost, and LightGBM algorithms. Dai and Shan et al. [29] studied an intelligent identification method for coal pillar stability in a fully mechanized caving face of thick coal seams. With a combination of finite difference methods, neural networks, and Monte Carlo simulation techniques, Li and Zhou et al. [30] studied underground mine hard rock pillar stability.

However, despite their reliable and accurate results, most algorithms are not easily applicable in practice due to their complex training and modeling procedures, and to their ‘black box’ features. The model tree algorithm—which jointly uses decision trees and linear regression methods–was proposed by Quinlan [31] to overcome such limitations. This algorithm has been successfully applied to numerous geotechnical problems, such as UCS and Young’s Modulus estimation [32] and hydraulic conductivity [33]. However, one of the key obstacles to using tree algorithms for pillar stability evaluation is that outcomes should be discrete values such as ‘stable’, ‘unstable’ or ‘failed’. Therefore, those discrete targets must be transformed into continuous values, so that they can be trained using a model tree algorithm for regression analysis.

We propose a Logistic Model Trees (LMT) method [34] to predict rock and coal pillar stability. This algorithm has the advantage of dealing with the classification problem by jointly using a tree model and a logistic regression algorithm, making it a rational choice in classification and decision-making. One main application of the LMT in geotechnical engineering has been landslide susceptibility prediction [35,36,37], but it has not yet been applied to predict underground pillar stability.

**Figure 1:**
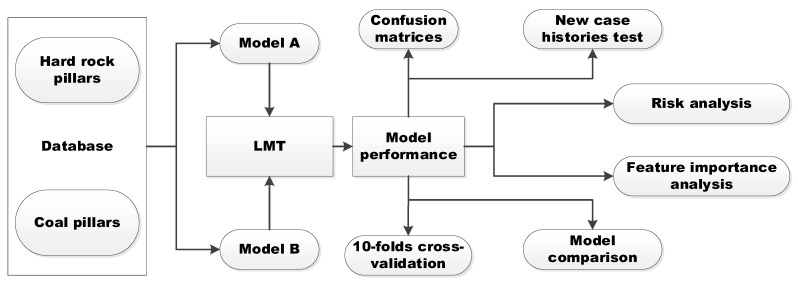
Flowchart of pillar stability prediction based on Logistic Model Trees (LMT).

The flowchart of this research is shown in Figure 1. The rest of this paper is organized as follows. Section 2 discusses two databases employed in this research, and the selection of input parameters. The LMT method is briefly introduced in Section 3. Section 4 discusses the performance and validation of proposed models, and risk and feature importance analyses.

## 2. Database Description

We compiled two databases that include recorded rock and coal pillar stability events from different types of underground mines. Database A was developed using observations collected by Lunder [6]. It includes 178 case histories (60 stable, 50 unstable, and 68 failed cases) that came from six hard rock mines in Canada, South Africa, and Sweden. Five main parameters—pillar width, *w*; pillar height, *h*; ratio of pillar width to height, *r*; uniaxial compressive strength of rock, UCS; and average pillar stress, *p*—that could affect rock pillar stability were selected and analyzed.

Similarly, Database B was developed based on Van der Merwe [38] and Van der Merwe [39], who provided information on 351 case histories of coal pillars (including 274 stable pillars and 77 failed cases) in South African coal mines. It originally contains four parameters related to coal pillar stability analyses —underground depth, H; pillar width, *w*; pillar height (mining height), *h*; bord width, B—so the ratio of pillar width to height (*r*) has been computed to be employed as a parameter in this database.

Although there are other features that could affect the rock and coal pillar stability, such as the geology structure conditions, water and gas contents, other stresses, and fracture information from CT or SEM images [14,40,41,42], that information is not available for the current pillar stability analysis. Since our models are trained using a limited number of data points are empirical methods, it is expected that their predictions are improved when a more extensive data set and/or more features are employed. Thus, a better calibration of model parameters, and more elaborate models could be established.

**Figure 2:**
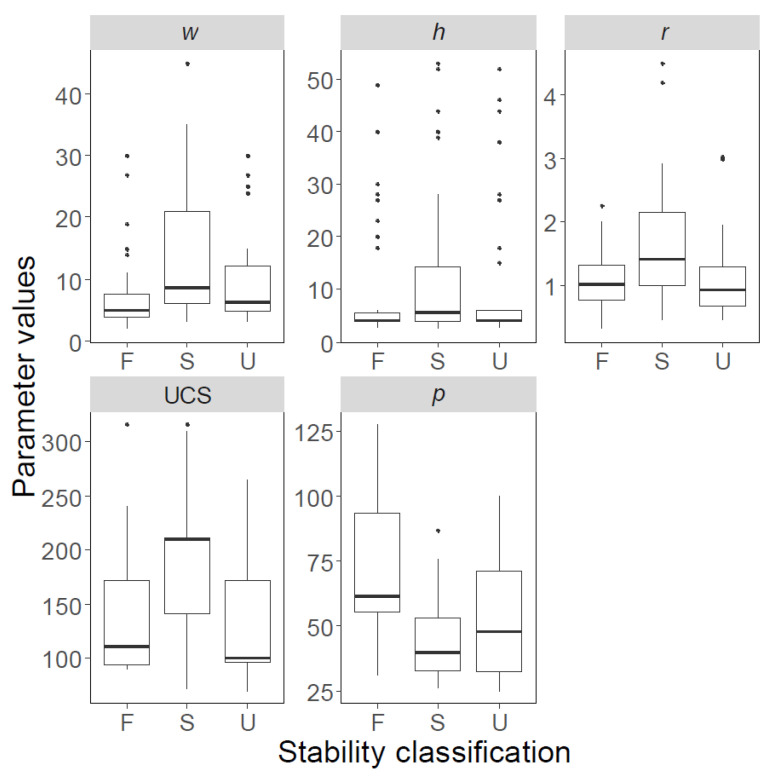
Variability of data points in Database A, presented as boxplots. F: failed; S: stable; U: unstable.

**Figure 3:**
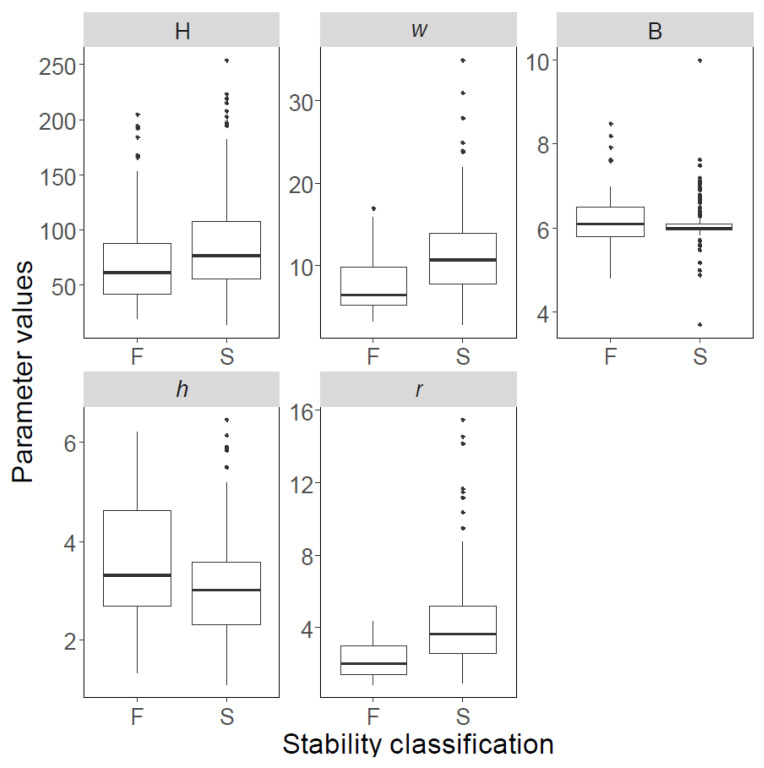
Variability of data points in Database B, presented as boxplots. F: failed; S: stable.

The boxplots of both databases are shown in Figure 2; Figure 3, and their statistical features are listed in Table 2; Table 3; solid black spots in Figure 2; Figure 3 denote “outliers”. The first and third quartiles are indicated using horizontal lines at the bottom and top of the boxes, whereas the bold lines inside the boxes represent median values. Similarly, pillars with ‘failed’, ‘stable’, and ‘unstable’ conditions are shown separately, and labelled using F, S, and U, respectively.

Based on the review and analysis of common techniques to estimate pillar stability using *FoS*, two categories of parameters were employed in our analyses. One category is the “strength” related parameters: pillar width, *w*; pillar height or mining height, *h*; ratio of pillar width to height, *r*; and uniaxial compressive strength of rock, UCS. The other category includes parameters related to the applied “stress,” including average pillar stress, *p*; underground depth, H; and bord width, B. With the availability of parameters for the two databases, Models 1 (*w*, *h*, *r*, UCS and *p*) and 2 (*w*, *h*, H, B and *r*) were established. The detailed analyses of these parameters are discussed in the following subsections.

### 2.1. Strength-Related Parameters

Pillar width *w* and pillar height *h* are two key geometrical parameters for stability analysis. The empirical approaches (linear, power, or shape forms) in Table 1 all included *w*, *h*, and *r* for pillar strength calculation. Zhou and Li et al. [2] employed *w*, *h*, *r*, and UCS as inputs for FDA and SVM models; Wattimena and Kramadibrata et al. [23] and Wattimena [4] used *r* as one of the parameters for regression analysis. Moreover, pillar shape, as represented by the *r* parameter, could have an influence on increased strength [3]. Therefore, we consider pillar width *w*, pillar height *h*, the ratio *r*, and UCS as “strength”-related parameters for Model 1. As Database B does not include UCS values, only *w*, *h*, and *r* are employed for Model 2.

### 2.2. Stress-Related Parameters

The actual stress acting on a pillar may depend on multiple factors such as in situ stresses, mining induced stress changes, geological features, shape and orientation of pillars, spatial relationship between pillars and mine openings, and groundwater conditions [6]. The tributary area theory introduced by Bunting [43] calculates the average stress acting on a pillar by simply supposing that the pillar supports its “share” of applied load. Salamon and Munro [11] proposed a more simplified equation:

(1) $$p=\gamma H{\left(\frac{w+B}{w}\right)}^{2}$$

where *γ* is the unit weight of the rock mass, H is underground depth, B is bord width between pillars, and *w* is pillar width.

For Model 1, the average stress has been calculated using the tributary area theory and two-dimensional or three-dimensional finite element modeling in the database, so that it can be directly applied for prediction. For Model 2, H, B, and *w* are employed to compute the stress condition for coal pillar stability.

Table 4 lists the selected parameters for each model, their corresponding data availability, and types. Theoretically, there may be additional indicators or parameters, such as geological condition, pillar orientation, or mining methods, that could have an influence on pillar stability; however, collecting these data is a major challenge and they have therefore not been employed in this work.

## 3. Logistic Model Trees

Logistic regression is a simple prediction tool, with advantages such as stability, low variance, and time-efficient training [44], but its prediction results are often biased. Decision trees are another machine learning method for searching a less restricted space of candidate models and capturing nonlinear patterns in a database; they exhibit low bias but high variance and instability, and are hence prone to overfitting. Therefore, the Logistic Model Trees (LMT) approach was proposed by Landwehr and Hall et al. [34]. It builds from the Model Tree approach proposed by Quinlan [31] to deal with regression problems with the joint use of linear regression and decision tree models, and is extended to classification problems. A brief introduction to LMT is presented in this section, while a more detailed description can be found in the seminal work of Landwehr and Hall et al. [34].

### 3.1. Tree Structure

**Figure 4:**
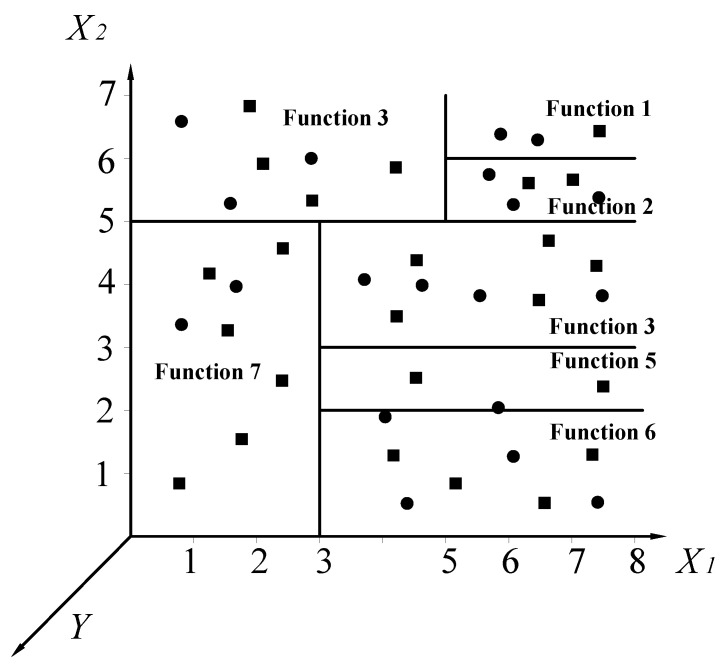
Example of input space split into seven subspaces (solid circle and square points represent different classes within one case, and each subspace has its own function).

A LMT includes a standard decision tree structure with logistic regression functions generated at the leaves. It has a set of inner or non-terminal nodes *N* and a set of leaves or terminal nodes *T*. Unlike a common decision tree or model tree, each leaf *t* of a LMT model has correlative logistic regression (LR) functions, instead of having classification labels or linear regression functions. For instance, suppose that the input vector *X* is (*X*_1_, *X*_2_) and the output target is *Y*. The whole instance space is denoted as *S*, and such space can be divided into several subspaces *S_t_*_._ A simple input space split into seven subspaces is shown in Figure 4. We can note that:

(2) $$S=\underset{t\in T}{\cup }S_{t},S_{t}\cap S_{t^{\prime }}=\emptyset$$

**Figure 5:**
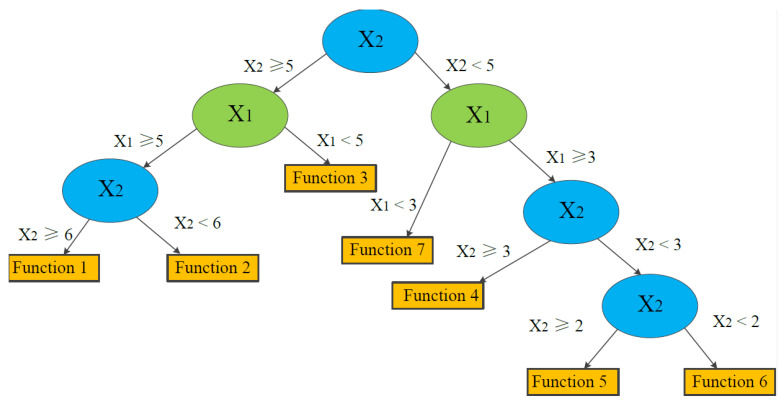
Example of a simplified tree structure of LMT associated with the example data in Figure 4 (modified from Landwehr and Hall et al. [34]).

For the seven divided subspaces in Figure 4, LR functions are determined in the model. The tree structure is shown in Figure 5.

### 3.2. Logistic Function

Unlike traditional forms of logistic regression, the LogitBoost algorithm for fitting additive logistic regression models proposed by Friedman and Hastie et al. [45] is employed here for model construction. The prediction probability is presented in Equation (3).

(3) $$\mathrm{Pr}\left(G=j|X=x\right)=\frac{e^{F_{j}\left(x\right)}}{\sum_{k=1}^{J}e^{F_{k}\left(x\right)}}$$

where *G* is the output, *J* are the class labels, *X* are the inputs, and *F_j_* (*x*) are the functions to be trained in the leaves of the tree by the LMT, as:

(4) $$F_{j}\left(x\right)=\sum_{m=1}^{M}f_{mj}\left(x\right)=\alpha_{0}^{j}+\sum_{s\in s_{t}}^{}\alpha_{s}^{j}\cdot s$$

where *m* is the number of iterations, *f_mj_* are the functions of input variables, *α* are the intercepts and coefficients of the linear function, and *s* are the variables of the subset *S_t_* at the leaf *t*.

### 3.3. LMT Training

A LMT can be established with the following steps: initial tree growing, tree splitting and stopping, and tree pruning. In this section, the basic idea is introduced; the reader is referred to Landwehr and Hall et al. [34] for detailed information.

The M5P method commonly employed for tree growing can build a standard tree first; then, a logistic regression model can be obtained at every node [32,46]. As this approach merely trains the model using case histories at each node in isolation, without considering the surrounding tree structure, another approach—one that can incrementally refine logistic model fit at high levels—is employed, so that the LogitBoost algorithm can iteratively change *F_j_* (*x*) to increase the fit in a natural way [34]. The function *f_mj_* is added to *F_j_* by altering one of the coefficients in the function or bringing in another variable (see Equation (4)).

Therefore, first, a LR tree is built in the root using proper iteration numbers determined by cross-validation in the initial growing process. Next, using the C4.5 splitting law [31] to raise the purity of the classification variable, the tree begins to grow by resembling advisable subsets (*t*) from database (*S*) to the children nodes. In the children nodes, the logistic regression functions are built by running the LogitBoost algorithm with the consideration of logistic model, weights, and probability estimate performed in the last iteration at the parent node. Then, another splitting process is performed. Considering reliable model fitting, the tree will stop splitting when a node has less than 15 cases. After the tree is built, the tree pruning method is employed to trade off tree size and to reduce model complexity, while still maintaining predictive accuracy. After different pruning scheme experiments, Landwehr and Hall et al. [34] employed the CAERT pruning method [47] to make pruning decisions considering training error and model complexity. Following these three steps, logistic model trees can be established.

## 4. Results and Discussion

### 4.1. Development of LMT Models for Pillar Stability Prediction

We employed the WEKA (Waikato Environment for Knowledge Analysis) [48] software to build models using the two databases collected. Since some case histories in Database A are incomplete (see Table 2), 153 case histories—55 stable cases, 34 unstable cases, and 64 failed cases—were employed as the training set, and nine case histories not used for training were randomly selected for validation. For Database B, 266 stable cases and 73 failed cases were employed as the training set and 12 case histories were selected for the validation work.

The ratio of stable to failed case numbers in the training set of Model 2 is about 3.64, indicating that the distribution of those two classes requires a cost-sensitive approach to overcome this ‘lack of balance’ problem [49,50]. Therefore, we consider the cost during the training process of Model 2. The cost-sensitive classifier is matched with the LMT algorithm using WEKA. As Model 2 is a binary prediction problem, a 2 × 2 cost matrix, shown in Table 5, is utilized during the training step. The value of C (F|S) is set to 4, so that false positive cases are four times more likely than false negative cases.

**Figure 6:**
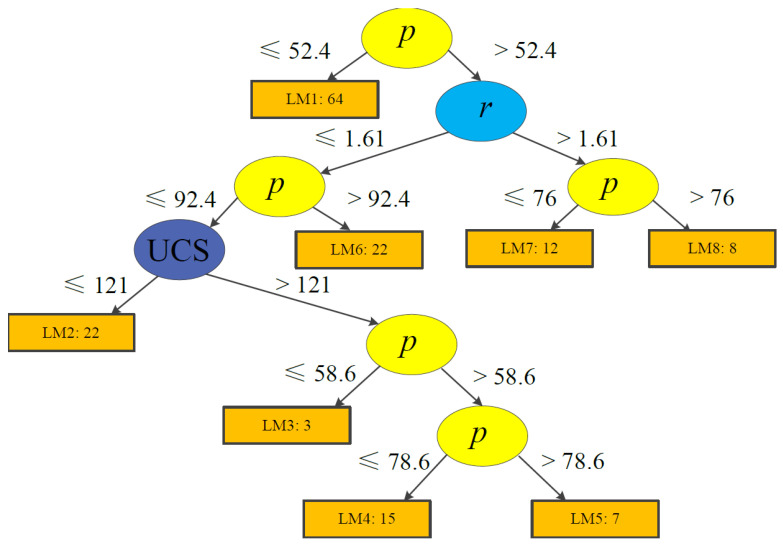
Structure of a logistic model tree for Model 1.

**Figure 7:**
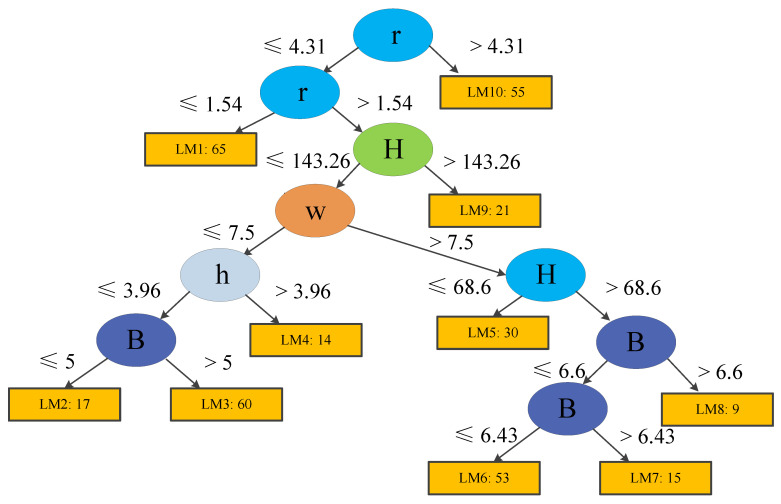
Structure of a logistic model tree for Model 2.

During tree model training, each terminal node (or leaf) is trained and updated using logistic regression models (see Section 3). Considering predictive performance, easily applicable tree structures, and the total number of training case histories, the minimum number of instances were set to 15 and 40 for Models 1 and 2, respectively. The trees produced using LMT are demonstrated in Figure 6; Figure 7. Model 1 contains eight logistic functions (LMs), whereas Model 2 includes ten LMs. The detailed expressions are listed in Table 6; Table 7.

As the variance inflation factor (VIF) can measure the correlation and strength of correlation between the variables in a regression model, we used R programing (car package) for the calculation of the VIF value; the results are as follows.

VIF > 10 indicates a potential multicollinearity problem in the dataset [35]. All the VIF values in Table 8 are smaller than 10, which is acceptable for regression analysis.

It should be noted that some functions in Table 6; Table 7 do not include all the parameters selected. For instance, the LM3 function for unstable pillars in Table 6 does not consider UCS. In the LMT training step, the simple logistic method is employed [34]. The aim of the simple logistic method is to control the parameter numbers, and to keep the model as simple and easy as possible. Therefore, new parameters are added, step by step, to improve the performance of each function at each node in the tree during training (see Section 3). This can also avoid the problem of model significance in the logistic regression, especially in the multiple logistic functions that build a full logistic model with all parameters. However, only some of the functions have less parameters than selected, indicating that most of our selected parameters affect predictive performance.

### 4.2. Model Performance and Validation

#### 4.2.1. Confusion Matrices

Three classes (stable, unstable, and failed) are predicted for case histories in Model 1, and two classes (stable and failed) for Model 2. Using all the LMs trained in Models 1 and 2, the confusion matrices that show predictive results are presented in Table 9; Table 10.

Many metrics can be employed to assess the predictive results of classification problems. We employed accuracy and Cohen’s Kappa values for the evaluation herein. For a confusion matrix with *n* rows and columns, *i* is the row counter, *j* is the column counter, and *m* is the element in the matrix. The accuracy can be computed as [51]:

(5) $$Ac=\left(\frac{\sum_{i=1}^{n}m_{ii}}{\sum_{i,j=1}^{n}m_{ij}}\right)\times 100\%$$

The Cohen’s Kappa coefficient can also be employed to assess predictive performance. However, it can only be employed on binary prediction results, so only Model 2 is evaluated by this metric. It is a robust measure of the proportion of cases classified correctly after the probability of chance agreement has been considered and removed [52]. The expression is [53]:

(6) $$\kappa =\frac{Ac-p_{e}}{1-p_{e}}$$

where *Ac* is the accuracy value obtained in Equation (5), and *P_e_* is the hypothetical probability of chance agreement, defined as Equation (7).

(7) $$p_{e}=\frac{\sum_{i=1}^{n}m_{i+}\cdot m_{+i}}{{\left(\sum_{i,j=1}^{n}m_{ij}\right)}^{2}}$$

where *m_i+_* and *m _+ i_* are the sum of elements in the *i*-th row and *i*-th column of the confusion matrix. The results of *κ* reflect the agreement condition of the results, with a complete agreement corresponding to a value of 1 and no agreement corresponding to less than 1.

The accuracy for Model 1 was equal to 94.1%, showing a satisfying predictive performance. For Model 2, accuracies for the training results with and without cost matrix were 92.9% and 93.1%, respectively, which conform to Kappa coefficients of 0.80 and 0.79. Both metrics indicate that the predictive results are reliable and acceptable.

#### 4.2.2. Cross-Validation

A 10-fold cross-validation procedure was adopted to validate Models 1 and 2 and to further examine their predictive results. For 10-fold cross-validation, each database was randomly sorted into 10 datasets; then, for each dataset, the remaining nine datasets were used to train the model, and the initially selected set was used for pillar stability evaluation with the trained LR models and to examine their performance using the observations within the set. The process was then repeated for all the ten sets. Results are listed in Table 11; Table 12, revealing that the average accuracy values for Models 1 and 2 are 79.1% and 80.5%, respectively.

#### 4.2.3. Validation through New Cases

Validation of model performance can also be conducted using new case histories. Therefore, another nine and twelve case histories (for Models 1 and 2, respectively) that were not employed for training work were used for validation. Their observed pillar stability conditions, as well as the predictive results obtained with functions in the tree models, are reported in Table 13; Table 14, where one can observe that our proposed tree models performed very well for most cases. For Model 1, all the case histories are well predicted. For Model 2, only three cases out of 12 are wrongly predicted. The test results confirm that the two proposed logistic tree models can reliably predict new case histories.

### 4.3. Comparison

To further assess our proposed Models 1 and 2, the results of several machine learning methods that have been studied in the literature, and of some other techniques that have not yet been employed for pillar stability prediction, are compared with the results of our models.

Table 15 compares prediction results for Model 1. It should be noted that although all the models proposed in the literature achieved good predictions with different input parameters, our proposed model has superior predictive performance. We can also note that the random forests shows the optimal predictive performance among all techniques (Techniques No. 6 to No. 12 are trained by WEKA); however, its cross-validation result is lower than the model proposed in this research. In other words, our proposed Model 1 has better predictive performance in both the training and cross-validation stages.

No machine learning techniques have been yet presented in the literature to predict coal pillar stability based on Database B. Therefore, for the assessment of Model 2, we merely compared several popular machine learning techniques trained by WEKA. The results are listed in Table 16. Note that fandom forests, decision trees and our proposed LMT show better predictive performance than the other methods considered; however, when compared to the other two algorithms (random forest and decision trees), our proposed model has the advantage that it can also be utilized for probabilistic risk analyses (discussed in the next section).

In addition, the main advantage of the proposed Models 1 and 2 is that they illustrate an explicit class probability using input parameters, which can be easily employed to predict the stability and potential risk of pillars using simple calculation by engineers. (The developed MATLAB “m-files” are provided in the Supplementary Material File S1). However, in the “black box” nature of other machine learning techniques, the training and predicting procedures can only be accomplished using other tools such as WEKA, R, or MATLAB. Therefore, they cannot be easily applied for quick forecast directly. For instance, the random forests algorithm may predict pillar stability more accurately, but it does not provide any equations that can be used for such evaluation.

### 4.4. Risk Analysis

We use two case histories in Table 13; Table 14 to present an application of the proposed Models 1 and 2 for risk analyses. The group of typical input data for Case 1 is as follows: *w* = 3.5 m, *h* = 3.8 m, *r* = 0.92, UCS = 94 MPa, and *p* = 55 MPa; for Case 2, we have H = 106.68 m, *w* = 12.19 m, B = 6.1 m, and *h* = 4.27 m. Then, given the tree structures from Figure 3; Figure 4, we employ the proper functions to calculate the probability of failure (*PoF*). For instance, as the value of *p* for Case 1 is 55 MPa, we take the right branch of the tree for Model 1, and then we check the *r* value. With the *r* value being equal to 0.92, we turn to the left branch and compare the *p* value again; then the final function (LM2) for this case is decided by the UCS value of 94 MPa. According to the LM2 equations in Table 5, we first calculate function values (F_S_, F_U_, and F_F_); then, using Equation (3), the probability of rock pillar stability can be expressed by Equation (11):

(8) $$F_{s}=–0.29+0.07w-0.21h+0.07r+0.06\mathrm{UCS}-0.39p$$

(9) $$F_{U}=97.07-1.26w-0.02h-7.19r-1.55p$$

(10) $$F_{F}=-93.43+1.18w+0.07h+4.74r-0.04\mathrm{UCS}+1.63p$$

(11) $$P_{S}=\frac{e^{F_{S}}}{e^{F_{S}}+e^{F_{U}}+e^{F_{F}}},P_{U}=\frac{e^{F_{U}}}{e^{F_{S}}+e^{F_{U}}+e^{F_{F}}},P_{F}=\frac{e^{F_{F}}}{e^{F_{S}}+e^{F_{U}}+e^{F_{F}}}$$

Finally, we can obtain the results: *Ps* = 0, *P_U_* = 37.8%, and *P_F_* = 62.2%, which implies that this case has a *PoF* equals to 62.2%. For Case 2 of Model 2, the function (LM6) in Table 6 can be used to calculate the PoF using the same method. Results show that the probability of a stable coal pillar for Case 2 is 91.1%, which also agrees with the actual conditions. Such probability results can be incorporated into risk analyses with the corresponding failure cost assessment later.

### 4.5. Feature Importance Analyses

In the training process of Models 1 and 2, information gain measures the separation of the training cases according to their target classification by a given attribute. It is employed to select among the candidate features during the tree growing procedure. We suppose that a case history is (*x*, *y*) = (*x*_1_, *x*_2_, *x*_3_,..., *x*_k_, *y*) and *x*_k_ is the *k*-th attribute of that case with the corresponding class label *y*. The expected information gain is the change in information entropy *H* from an initial state to a state that takes some information, computed as [54]:

(12) $$G\left(S,k\right)=H\left(S\right)-\sum_{t\in value\left(k\right)}\frac{\left|S_{t}\right|}{\left|S\right|}H\left(S_{t}\right)$$

where *S* denotes the training data set and *S_t_* is the subset of *S* for which feature *k* has value of *t* ($S_{t}=\left\{x\in S|x_{k}=t\right\}$).

In the tree model training, before determining the feature in the root node, all the information gains provided by different features are calculated and compared, and the most influential feature is chosen as the root node in the tree structure. The other features in the nodes of the decision tree appear in descending order of importance [32,55]. Compared to other machine learning techniques, for which extra analyses are required to analyze sensitivity, the LMT decides the most influential feature during the tree growing process. Therefore, the average stress *p* is the most significant parameter in the pillar stability prediction for Model 1, followed by the ratio of pillar width to height *r*. Similarly, the most critical parameter for Model 2 is also *r*, followed by the excavation depth H. Both importance analyses show that pillar stability is largely influenced by the applied stress, ratio of width to height, and depth, thus agreeing with the empirical observations mentioned earlier in this paper.

## 5. Conclusions

A novel application of Logistic Model Trees (LMT) for instability assessment of underground pillars is presented. Tree structures and the corresponding functions are employed to assess the stability of pillars, given information on several features—including pillar width, pillar height, ratio of pillar width to height, uniaxial compressive strength of rock, average pillar stress, underground depth, and bord width—based on which predictions are conducted. The LMT is learned by LogitBoost, using two sets of case histories from the databases that we compiled from the literature. Results show that both Models 1 and 2 can accurately predict the stability of rock and coal pillars.

The trained tree models were validated using the original databases and 10-fold cross-validation. In addition, nine and twelve new cases—not previously employed for training—were used to further validate the models proposed. Moreover, predictions of several other machine learning methods were also compared with our proposed models. Results showed that the accuracies of the proposed tree models are among the highest and that they are probably adequate for mine site applications. In addition, results suggest that the LMT technique can offer useful information about the probability of pillar failure during underground excavation, so it can be utilized for risk analyses of pillar stability.

Furthermore, feature importance analyses were conducted to appraise the most influential input features for pillar stability. The average pillar stress *p* was found as the most influential parameter for Model 1, with other factors such as *r* and UCS also having a critical influence on predictions. For Model 2, *r* showed the highest effect on the output, followed by H and *w*. It is expected that these results can lead mine engineers to focus their efforts towards those crucial identified factors.

As the dataset employed for LMT learning and training was limited, it is expected that its predictive capacity could be increased if more case histories are collected. In addition, there are a few minor influencing parameters such as pore-water pressure, gas and water contents of the rocks, etc., which are neglected in this study due to the unavailability of data, and could have been among the selected parameters. More importantly, the application of the proposed models should be within the range of selected parameters.

Finally, the main advantage of LMT compared to other related algorithms commonly employed for pillar stability estimation is that it can be trained easily (even with more input parameters) and that its tree structure, with a LR function in each leaf, explicitly demonstrates the relationship between the inputs and predictive outputs. In addition, it is expected that, with its intuitive features and easy implementation, the LMT can also be employed to solve other geotechnical problems in the future.

## Figures and Tables

**Table 1:** Typical empirical methods for estimating of pillar strength.

Reference	Equation	Form
Obert and Duvall [8]	Ps = UCS (0.778 + 0.222(w/h))	Linear shape effect
Bieniawski and Van Heerden [9]	Ps = UCS (0.64 + 0.34(w/h))	Linear shape effect
Hoek and Brown [10]	σ_1_ = σ_3_ + ((mσ_c_σ_3_) + (sσ_c_))^0.5^	Hoek-Brown
Salamon and Munro [11]	Ps = UCSw^0.46^/h^0.64^	Size effect
Bieniawski [12]	Ps = UCSw^0.16^/h^0.55^	Size effect
Galvin and Hebblewhite et al. [13]	Ps = UCSw^0.50^/h^0.70^	Size effect
Lunder [6]	Ps = UCS (w/h)^0.5^	Power shape effect
Lunder [6]	Ps = 0.44UCS(0.68 + 0.52κ), with κ = tan(cos^−1^((1−C_pav_)/(1 + C_pav_))) and C_pav_ = 0.46 (log(w/h + 0.75))^1.4/(w/h)^	Confinement
Prassetyo and Irnawan et al. [14]	Ps = 3.2 (0.14 + 0.86(w/h)) Ps = 2.7 (0.12 + 0.88(w/h))	Linear shape effect
Prassetyo and Irnawan et al. [14]	Ps = 3.7 (w/h)^0.9^ Ps = 3.4 (w/h)^0.8^	Power shape effect

Notation: Ps, pillar strength (MPa); UCS, unconfined compressive strength of a cubic pillar specimen (MPa); w, pillar width (m); h, pillar height (m); σ_1_, major principal stress (MPa); σ_3_, Minor principal stress (MPa); σ_c_, unconfined compressive strength of intact rock (MPa); m and s, empirically derived constants based on rock mass quality of pillar material; κ, pillar confinement/friction factor; C_pav_, average σ_3_/σ_1_ ratio across the mid-height centerline of the pillar.

**Table 2:** Statistical values of features for case histories in Database A.

Parameter	Available	Missing	Min	Max	Mean	Standard Deviation
*w* (m)	162	16	1.9	45	10.16	8.76
*h* (m)	162	16	2.4	53	10.38	12.40
*R*	178	0	0.31	4.5	1.25	0.66
UCS (MPa)	178	0	70	316	155.99	61.73
*p* (MPa)	178	0	25	127.6	57.30	23.33

**Table 3:** Statistical values of features for case histories in Database B.

Parameter	Available	Missing	Min	Max	Mean	Standard Deviation
H (m)	351	0	13.22	254.45	83.11	44.59
*w* (m)	351	0	2.74	35	10.61	5.03
B (m)	351	0	3.69	10	6.09	0.61
*h* (m)	351	0	1.1	6.45	3.12	1.12
*r*	351	0	0.87	15.45	3.81	2.26

**Table 4:** Parameter selection for each model.

Parameters	Model 1	Model 2
Pillar width, *w* (m)	√	√
Pillar height, *h* (m)	√	√
Ratio of *w* to *h*, *r*	√	√
UCS (MPa)	√	
Average pillar stress, *p* (MPa)	√	
Underground depth, H (m)		√
Bord width, B (m)		√

**Table 5:** Cost matrix of pillar stability prediction with Model 2.

Predicted			
Stability	Failure		
0	1	Stability	Actual
4	0	Failure	

**Table 6:** Logistic functions for Model 1.

No.	Stability Condition	Regression Models
LM1	S	F_1_ = −0.91 + 0.06*w* − 0.02*h* + 1.61*r* + 0.03UCS − 0.12*p*
	U	F_2_ = 3.03 − 0.06*w* + 0.04*h* + 0.14*r −* 0.07*p*
	F	F_3_ = 10.15 − 0.01*w* + 0.01*h* − 18.09*r* − 0.03UCS + 0.25*p*
LM2	S	F_1_ = −0.29 + 0.07*w* − 0.21*h* + 0.07*r* + 0.06UCS − 0.39*p*
	U	F_2_ = 97.07 − 1.26*w* − 0.02*h* − 7.19*r* − 1.55*p*
	F	F_3_ = −93.43 + 1.18*w* + 0.07*h* + 4.74*r* − 0.04UCS + 1.63*p*
LM3	S	F_1_ = 18.93 + 0.12*w* − 0.28*h* − *r* + 0.06UCS − 0.47*p*
	U	F_2_ = 5.39 − 0.01*w* − 0.03*h* − 3.02*r*
	F	F_3_ = −3.66 − 0.07*w* + 0.1*h* + 1.07*r* − 0.02UCS + 0.09*p*
LM4	S	F_1_ =17.65 + 0.29*w* − 0.42*h* − 3.21*r* + 0.06UCS − 0.4*p*
	U	F_2_ = −1.05 − 0.05*h* − 2.34*r* + 0.08*p*
	F	F_3_ = 46.91 − 0.45*w* + 0.15*h* − 35.86*r* − 0.06UCS − 0.26*p*
LM5	S	F_1_ = 8.22 + 0.21*w* − 0.35*h* − 2.3*r* + 0.06UCS − 0.47*p*
	U	F_2_ = −59.67 − 0.13*w −* 0.08*h* − 17.17*r* +0.11UCS + 0.64*p*
	F	F_3_ = 63.53 − 0.04*w* + 0.15*h* + 15.07*r* − 0.14UCS − 0.57*p*
LM6	S	F_1_ = −3.59 + 0.03*w* − 0.15*h* + 0.93*r* + 0.06UCS − 0.33*p*
	U	F_2_ = −8.93 − 0.03*w* − 0.06*h* − 1.58*r* − 0.03*p*
	F	F_3_ = 12.17 − 0.03*w* + 0.11*h* − 1.06*r −* 0.03UCS + 0.11*p*
LM7	S	F_1_ = −27.03 + 0.26*w* − 0.02*h* + 9.89*r* + 0.12UCS − 0.18*p*
	U	F_2_ = 352.78 − 0.2*w* + 0.02*h* − 0.89*r* − 0.03UCS − 6.35*p*
	F	F_3_ = 15.48 − 0.17*w* − 0.01*h* − 5.85*r* − 0.08UCS + 0.13*p*
LM8	S	F_1_ = 10.24 − 0.29*w* − 12.14*h* + 6.82*r* + 0.2UCS − 0.25*p*
	U	F_2_ = 20.3 + 1.02*w* + 0.02*h* + 15.21*r* − 0.15UCS − 0.29*p*
	F	F_3_ = 42.74 − 0.77*w* − 0.01*h* − 48.27*r* + 0.02UCS + 0.51*p*

**Table 7:** Logistic functions for Model 2.

No.	Stability Condition	Regression Models
LM1	S	F_1_ = −8.58 − 0.03H + 1.22*w* + 0.102B− 0.11*h* + 0.51*r*
	F	F_2_ = −F_1_
LM2	S	F_1_ = 498.57 −0.04*w* − 101.31B + 0.59*h* + 0.32*r*
	F	F_2_ = −F_1_
LM3	S	F_1_ = 2.1 − 0.03H + 0.59*w* − B + 0.59*h* + 0.63*r*
	F	F_2_ = −F_1_
LM4	S	F_1_ = −6.11 − 0.01H + 0.03*w* + 0.02B − 0.11*h* + 0.16*r*
	F	F_2_ = −F_1_
LM5	S	F_1_ = 5.75 − 0.02H + 0.15B + 0.09*h* + 0.2*r*
	F	F_2_ = −F_1_
LM6	S	F_1_ = −8.04 + 0.01H + 0.01*w* + 1.15B + 0.16*h* + 0.11*r*
	F	F_2_ = −F_1_
LM7	S	F_1_ = −6.01 − 0.02*w* − 0.19B + 0.2*h* + 0.19*r*
	F	F_2_ = −F_1_
LM8	S	F_1_ = 3.41 − 0.04*w* + 0.32B + 0.16*h* + 0.2*r*
	F	F_2_ = −F_1_
LM9	S	F_1_ = −7.2 − 0.02H + 0.12*w* + 0.15B + 0.03*h* + 0.27*r*
	F	F_2_ = −F_1_
LM10	S	F_1_ = 3.4 − 0.01H + 0.09*w*+ 0.12B + 0.53*r*
	F	F_2_ = −F_1_

**Table 8:** The VIF values for parameters in Models 1 and 2.

Parameters	Model 1	Model 2
Pillar width, *w* (m)	5.57	4.81
Pillar height, *h* (m)	6.62	2.94
Ratio of *w* to *h*, *r*	2.27	6.71
UCS (MPa)	1.33	
Average pillar stress, *p* (MPa)	1.40	
Underground depth, H (m)		2.71
Bord width, B (m)		1.14

**Table 9:** Confusion matrix of rock pillar prediction with Model 1.

Predicted				
Stable	Unstable	Failed		
53	2	0	Stable	Actual
4	29	1	Unstable	
1	1	62	Failed	

**Table 10:** Confusion matrix of coal pillar prediction with Model 2.

Predicted without cost matrix			
Stable	Failed		
258	8	Stable	Actual
15	58	Failed	
Predicted with cost matrix			
Stable	Failed		
251	15	Stable	Actual
9	64	Failed	

**Table 11:** Confusion matrices of Model 1 by 10-fold cross-validation.

Validation Group	Accuracy	Predicted	Confusion Matrices		
		Stable	Unstable	Failed	Actual
No.1	56.3%	3	2	0	Stable
		2	0	2	Unstable
		0	1	6	Failed
No.2	93.8%	5	0	0	Stable
		0	3	1	Unstable
		0	0	7	Failed
No.3	87.5%	4	1	0	Stable
		0	4	0	Unstable
		1	0	6	Failed
No.4	86.7%	5	0	0	Stable
		0	1	2	Unstable
		0	0	7	Failed
No.5	80%	5	1	0	Stable
		0	1	2	Unstable
		0	0	6	Failed
No.6	80%	4	1	1	Stable
		1	2	0	Unstable
		0	0	6	Failed
No.7	73.3%	6	0	0	Stable
		1	1	1	Unstable
		1	1	4	Failed
No.8	73.3%	5	1	0	Stable
		1	0	2	Unstable
		0	0	6	Failed
No.9	86.7%	6	0	0	Stable
		0	2	1	Unstable
		0	1	5	Failed
No.10	73.3%	5	0	0	Stable
		2	1	1	Unstable
		0	1	5	Failed
Average	79.1%				

**Table 12:** Confusion matrices of Model 2 by 10-fold cross-validation.

Validation Group	Accuracy	Confusion Matrices		
		Predicted		
		Stable	Failed	Actual
No.1	85.3%	23	4	Stable
		1	6	Failed
No.2	58.8%	17	10	Stable
		4	3	Failed
No.3	79.4%	22	5	Stable
		2	5	Failed
No.4	85.3%	22	5	Stable
		0	7	Failed
No.5	94.1%	25	2	Stable
		0	7	Failed
No.6	91.2%	25	2	Stable
		1	6	Failed
No.7	76.5%	21	5	Stable
		3	5	Failed
No.8	76.5%	21	5	Stable
		3	5	Failed
No.9	82.3%	22	4	Stable
		2	6	Failed
No.10	75.6%	19	7	Stable
		1	6	Failed
Average	80.5%			

**Table 13:** Predicted results of nine new cases with Model 1.

No.	*h* (m)	*w* (m)	*r*	UCS (MPa)	*p* (MPa)	Observed	Predicted Results
1	3	3	1	210	44.1	S	S
2	6.1	5.5	1.1	210	26.2	S	S
3	12	8	1.5	215	28	S	S
4	5.7	3.8	1.5	94	47	U	U
5	6.3	3.8	1.66	94	48	U	U
6	5.3	3.8	1.39	94	48	U	U
7	4.6	3.8	1.21	94	63	F	F
8	4.6	3.8	1.21	94	54	F	F
9	3.5	3.8	0.92	94	55	F	F

**Table 14:** Predicted results of twelve new cases with Model 2.

No.	H (m)	*w* (m)	B (m)	*h* (m)	*r*	Observed	Predicted Results
1	219.46	21.73	5.58	3.17	6.85	S	S
2	114	17.37	5.49	1.98	8.77	S	S
3	106.68	12.19	6.1	4.27	2.85	S	S
4	198.12	17.16	5.7	2.83	6.06	S	S
5	76.2	7.62	6.1	4.57	1.67	S	S
6	182.88	15.85	5.49	4.88	3.25	S	F
7	182.88	16.92	5.94	2.44	6.93	S	S
8	91.44	12.19	6.1	1.52	8.02	S	S
9	19	6	6	3	2.00	F	S
10	21.3	4	8.2	4.6	0.87	F	F
11	22	3.5	6.5	1.6	2.19	F	F
12	23	6	6	2.9	2.07	F	S

**Table 15:** Comparison of Model 1 with other ML techniques reported in previous works and trained by WEKA.

No.	Techniques	Input Parameter	Case Histories	Accuracy Values	Average Accuracy (10-Fold CV)	References
1	Logistic regression	*r*, *p*/UCS	178	79.2%	NA	Wattimena [4]
2	J48 (decision tree)	*r*, *p*/UCS	178	84.8%	NA	Ghasemi and Kalhori et al. [19]
3	SVM	*r*, *p*/UCS	178	82.0%	NA	Ghasemi and Kalhori et al. [19]
4	SVM	*h*, *w*, UCS, *Ps*, *p*	177	84.3%	NA	Zhou and Li et al. [3]
5	ANN	*r*, UCS, *p*	177	80.3%	NA	Zhou and Li et al. [3]
6	Logistic regression	*h*, *w*, *r*, UCS, *p*	153	81.0%	75.8%	NA
7	SVM	*h*, *w*, *r*, UCS, *p*	153	73.4%	69.3%	NA
8	ANN	*h*, *w*, *r*, UCS, *p*	153	89.5%	80.4%	NA
9	Naïve Bayes	*h*, *w*, *r*, UCS, *p*	153	60.1%	55.6%	NA
10	Random forests	*h*, *w*, *r*, UCS, *p*	153	100%	76.5%	NA
11	J48 (decision trees)	*h*, *w*, *r*, UCS, *p*	153	92.8%	73.2%	NA
12	Logistic model trees	*h*, *w*, *r*, UCS, *p*	153	94.1%	79.1%	NA

**Table 16:** Comparison of the proposed Model 2 with other ML techniques computed by WEKA.

No.	Techniques	Input Parameter	Case Histories	Accuracy Values	Average Accuracy (10-Fold CV)
1	Logistic regression	H, *w*, B, *h, r*	339	76.1%	74.0%
2	SVM	H, *w*, B, *h, r*	339	64.9%	62.2%
3	ANN	H, *w*, B, *h, r*	339	81.7%	77.0%
4	Naïve Bayes	H, *w*, B, *h, r*	339	59.9%	59.6%
5	Random forests	H, *w*, B, *h, r*	339	99.1%	85.8%
6	J48 (decision trees)	H, *w*, B, *h, r*	339	92.9%	80.8%
7	Logistic model trees	H, *w*, B, *h, r*	339	92.9%	80.5%

## Data Availability

Not applicable.

## Supplementary Materials

The following are available online at https://www.mdpi.com/article/10.3390/ijerph19042136/s1, File S1: Developed MATLAB “m-files”.

## References

[1] Brady B.H.G., Brown E.T. (1985). Rock Mechanics for Underground Mining.

[2] Zhou J., Li X.B., Shi X.Z., Wei W., Wu B.B. (2011). Predicting pillar stability for underground mine using Fisher discriminant analysis and SVM methods. Trans. Nonferrous Met. Soc. China.

[3] Zhou J., Li X.B., Mitri H.S. (2015). Comparative performance of six supervised learning methods for the development of models of hard rock pillar stability prediction. Nat. Hazards.

[4] Wattimena R.K. (2014). Predicting the stability of hard rock pillars using multinomial logistic regression. Int. J. Rock Mech. Min..

[5] Dou L., Lu C., Mu Z., Gao M. (2009). Prevention and forecasting of rock burst hazards in coal mines. Min. Sci. Technol..

[6] Lunder P.J. (1994). Hard Rock Pillar Strength Estimation an Applied Empirical Approach. Ph.D. Thesis.

[7] Ahmad M., Al-Shayea N.A., Tang X.-W., Jamal A., Al-Ahmadi M.H., Ahmad F. (2020). Predicting the Pillar Stability of Underground Mines with Random Trees and C4.5 Decision Trees. Appl. Sci..

[8] Obert L., Duvall W.I. (1967). Rock Mechanics and the Design of Structures in Rock.

[9] Bieniawski Z.T., Van Heerden W.L. (1975). The significance of in situ tests on large rock specimens. Int. J. Rock Mech. Min. Sci. Geomech. Abstr..

[10] Hoek E., Brown E.T. (1980). Underground Excavations in Rock.

[11] Salamon M., Munro A. (1967). A study of the strength of coal pillars. J. South. Afr. Inst. Min. Metall..

[12] Bieniawski Z.T. (1968). The effect of specimen size on compressive strength of coal. Int. J. Rock Mech. Min. Sci. Geomech. Abstr..

[13] Galvin J.M., Hebblewhite B.K., Salamon M.D. (1999). University of New South Wales coal pillar strength determinations for Australian and South African mining conditions. Proceedings of the Second International Workshop on Coal Pillar Mechanics and Design.

[14] Prassetyo S.H., Irnawan M.A., Simangunsong G.M., Wattimena R.K., Arif I., Rai M.A. (2019). New coal pillar strength formulae considering the effect of interface friction. Int. J. Rock Mech. Min..

[15] York G. (1998). Numerical modelling of the yielding of a stabilizing pillar/foundation system and a new design consideration for stabilizing pillar foundations. J. South. Afr. Inst. Min. Metall..

[16] Mortazavi A., Hassani F.P., Shabani M. (2009). A numerical investigation of rock pillar failure mechanism in underground openings. Comput. Geotech..

[17] Li X., Li D., Liu Z., Zhao G., Wang W. (2013). Determination of the minimum thickness of crown pillar for safe exploitation of a subsea gold mine based on numerical modelling. Int. J. Rock Mech. Min..

[18] Martin C.D., Maybee W.G. (2000). The strength of hard-rock pillars. Int. J. Rock Mech. Min..

[19] Deng J., Yue Z.Q., Tham L.G., Zhu H.H. (2003). Pillar design by combining finite element methods, neural networks and reliability: A case study of the Feng Huangshan copper mine, China. Int. J. Rock Mech. Min..

[20] Elmo D., Stead D. (2010). An Integrated Numerical Modelling–Discrete Fracture Network Approach Applied to the Characterisation of Rock Mass Strength of Naturally Fractured Pillars. Rock Mech. Rock Eng..

[21] Tawadrous A.S., Katsabanis P.D. (2007). Prediction of surface crown pillar stability using artificial neural networks. Int. J. Numer. Anal. Met..

[22] Recio-Gordo D., Jimenez R. (2012). A probabilistic extension to the empirical ALPS and ARMPS systems for coal pillar design. Int. J. Rock Mech. Min..

[23] Wattimena R.K., Kramadibrata S., Sidi I.D., Azizi M.A. (2013). Developing coal pillar stability chart using logistic regression. Int. J. Rock Mech. Min..

[24] Ghasemi E., Ataei M., Shahriar K. (2014). An intelligent approach to predict pillar sizing in designing room and pillar coal mines. Int. J. Rock Mech. Min..

[25] Ghasemi E., Ataei M., Shahriar K. (2014). Prediction of global stability in room and pillar coal mines. Nat. Hazards.

[26] Ghasemi E., Kalhori H., Bagherpour R. (2017). Stability assessment of hard rock pillars using two intelligent classification techniques: A comparative study. Tunn. Undergr. Space Technol..

[27] Mohanto S., Deb D. (2020). Prediction of Plastic Damage Index for Assessing Rib Pillar Stability in Underground Metal Mine Using Multi-variate Regression and Artificial Neural Network Techniques. Geotech. Geol. Eng..

[28] Liang W., Luo S., Zhao G., Wu H. (2020). Predicting Hard Rock Pillar Stability Using GBDT, XGBoost, and LightGBM Algorithms. Mathematics.

[29] Dai J., Shan P., Zhou Q. (2020). Study on Intelligent Identification Method of Coal Pillar Stability in Fully Mechanized Caving Face of Thick Coal Seam. Energies.

[30] Li C., Zhou J., Armaghani D.J., Li X. (2021). Stability analysis of underground mine hard rock pillars via combination of finite difference methods, neural networks, and Monte Carlo simulation techniques. Undergr. Space.

[31] Quinlan J.R. (1992). Learning with continuous classes. Proceedings of the 5th Australian Joint Conference on Artificial Intelligence.

[32] Ghasemi E., Kalhori H., Bagherpour R., Yagiz S. (2016). Model tree approach for predicting uniaxial compressive strength and Young’s modulus of carbonate rocks. Bull. Eng. Geol. Environ..

[33] Naeej M., Naeej M.R., Salehi J., Rahimi R. (2017). Hydraulic conductivity prediction based on grain-size distribution using M5 model tree. Geomech. Geoengin..

[34] Landwehr N., Hall M., Frank E. (2005). Logistic Model Trees. Mach. Learn..

[35] Tien Bui D., Tuan T.A., Klempe H., Pradhan B., Revhaug I. (2016). Spatial prediction models for shallow landslide hazards: A comparative assessment of the efficacy of support vector machines, artificial neural networks, kernel logistic regression, and logistic model tree. Landslides.

[36] Chen W., Xie X., Wang J., Pradhan B., Hong H., Bui D.T., Duan Z., Ma J. (2017). A comparative study of logistic model tree, random forest, and classification and regression tree models for spatial prediction of landslide susceptibility. Catena.

[37] Chen W., Zhao X., Shahabi H., Shirzadi A., Khosravi K., Chai H., Zhang S., Zhang L., Ma J., Chen Y. (2019). Spatial prediction of landslide susceptibility by combining evidential belief function, logistic regression and logistic model tree. Geocarto Int..

[38] Van der Merwe J.N. (2003). New pillar strength formula for South African coal. J. South. Afr. Inst. Min. Metall..

[39] Van der Merwe J.N. (2006). South African coal pillar database. J. South. Afr. Inst. Min. Metall..

[40] Zhou Z., Zang H., Cao W., Du X., Chen L., Ke C. (2019). Risk assessment for the cascading failure of underground pillar sections considering interaction between pillars. Int. J. Rock Mech. Min..

[41] Zhang X., Nguyen H., Bui X.-N., Anh Le H., Nguyen-Thoi T., Moayedi H., Mahesh V. (2020). Evaluating and Predicting the Stability of Roadways in Tunnelling and Underground Space Using Artificial Neural Network-Based Particle Swarm Optimization. Tunn. Undergr. Space Technol..

[42] Finzi Y., Ganz N., Dor O., Davis M., Volk O., Langer S., Arrowsmith R., Tsesarsky M. (2020). Stability Analysis of Fragile Rock Pillars and Insights on Fault Activity in the Negev, Israel. J. Geophys. Res. Solid Earth.

[43] Bunting D. (1911). Chamber pillars in deep anthracite mines. Trans. AIME.

[44] Hosmer D.W., Lemeshow S., Sturdivant R.X. (2013). Applied Logistic Regression.

[45] Friedman J., Hastie T., Tibshirani R. (2000). Additive logistic regression: A statistical view of boosting (with discussion and a rejoinder by the authors). Ann. Stat..

[46] Wang Y., Witten I.H. Inducing Model Trees for Continuous Classes. Proceedings of the 9th European Conference on Machine Learning Poster Papers.

[47] Breiman L., Friedman J., Stone C.J., Olshen R.A. (1984). Classification and Regression Trees.

[48] Witten I.H., Frank E., Hall M.A., Pal C.J. (2016). Data Mining: Practical Machine Learning Tools and Techniques.

[49] Elkan C. (2001). The foundations of cost-sensitive learning. Proceedings of the International Joint Conference on Artificial Intelligence.

[50] Zazzaro G., Pisano F.M., Mercogliano P. (2010). Data Mining to Classify Fog Events by Applying Cost-Sensitive Classifier. Proceedings of the 2010 International Conference on Complex, Intelligent and Software Intensive Systems.

[51] Fawcett T. (2006). An introduction to ROC analysis. Pattern Recogn. Lett..

[52] Kuhn M., Johnson K. (2013). Applied Predictive Modeling.

[53] Cohen J. (1960). A Coefficient of agreement for nominal scales. Educ. Psychol. Meas..

[54] Mitchell T.M. (1997). Machine Learning.

[55] Sugumaran V., Muralidharan V., Ramachandran K.I. (2007). Feature selection using Decision Tree and classification through Proximal Support Vector Machine for fault diagnostics of roller bearing. Mech. Syst. Signal Process..

